# Prevalence, combination patterns, and quality of life factors of multimorbidity among older adults in southern China based on the health ecological model

**DOI:** 10.7189/jogh.15.04215

**Published:** 2025-07-25

**Authors:** Chunxiao Long, Jiaqi Huang, Di Liu, Can Liu, Mengting Wu, Haiyang Wu, Jun Deng, Yinjuan Zhang, Lei Shi, Yanze Cui

**Affiliations:** 1 School of Health Management, Guangzhou Medical University, Guangzhou, China; 2 School of Marxism, Harbin Medical University, Harbin, China; 3 The Second School of Clinical Medicine, Guangzhou Medical University, Guangzhou, China; 4 School of Medicine, Dali University, Dali, China; 5 School of Health Management, Southern Medical University, Guangzhou, China; 6 Philosophy and Social Sciences Key Laboratory of Guangdong Higher Education Institutes for Health Governance Based on Big Data Utilization, Guangzhou Medical University, Guangzhou, China; 7 Local Government Development Research Institute of Shantou University, Shantou, China; 8 The First Affiliated Hospital of Harbin Medical University, Harbin, China

## Abstract

**Background:**

Multimorbidity is increasingly prevalent among older adults and poses significant challenges to health and well-being. This study applied a health ecological model to investigate the prevalence, determinants, and common disease patterns of multimorbidity, as well as the factors associated with quality of life (QoL) among older adults in southern China.

**Methods:**

A cross-sectional survey was conducted among 2404 individuals aged 60 years and older using a multi-stage random sampling method. Quality of life was assessed using the EQ-5D-5L scale. Multimorbidity was defined as the presence of two or more chronic conditions. The Apriori algorithm identified common multimorbidity combinations. Factors influencing multimorbidity were analysed using univariate and multivariate logistic regression based on a health ecological model. Tobit regression was used to assess associated factors of QoL among patients with multimorbidity.

**Results:**

The prevalence of multimorbidity was 44.3%. Hypertension featured prominently in disease clusters, with ‘hypertension + hyperlipidemia’ as the top two-disease combination. Risk factors for multimorbidity included QoL, age, body mass index (BMI), exercise, sleep quality, social participation, education level, per capita monthly household income, and region. The number of chronic diseases was negatively associated with QoL. Factors significantly influencing QoL included age(≥80, β = −0.087, *P* < 0.001), number of chronic diseases(>3 diseases, β = −0.029, *P* = 0.012), fresh fruit intake (occasionally: β = 0.052; often: β = 0.064, all *P* < 0.005), dietary balance (always: β = 0.078, *P* = 0.007), exercise frequency (1–3 days: β = −0.039; >3 days: β = 0.024, all *P* < 0.005), sleep quality (better: β = −0.034; worse: β = −0.070; very bad: β = −0.161; all *P* < 0.005), social participation (β = 0.034; *P* = 0.006), education level (primary school: β = 0.028, *P* = 0.028; college/higher vocational school: β = 0.083, *P* = 0.010), and region (western: β = 0.083; northern: β = 0.064; eastern: β = 0.132; all *P* < 0.001).

**Conclusions:**

Multimorbidity among older adults in southern China is associated with demographic, behavioral, interpersonal, socioeconomic, and regional factors. Therefore, it is recommended to implement differentiated insurance reimbursement, reinforce county-level resource allocation, integrate community services via the World Health Organization’s (WHO) Integrated Care for Older People (ICOPE) framework, and promote individual lifestyle measures. Given the reliance on self-reported cross-sectional data, the findings are constrained by limited causal inference and possible recall bias. Longitudinal studies are needed to validate and refine the conclusions.

The global population is ageing rapidly. It is projected that the population aged 60 and over will double between 2013 and 2050, a trend that has drawn widespread attention to the challenges of population aging [1]. China is home to 20% of the world's older adults and is facing a particularly serious ageing challenge. By the end of 2024, it is estimated that China’s older adults reached 310 million, accounting for 22% of its total population and 4% of the global population [2,3]. Population ageing has become a fundamental demographic trend in contemporary society, significantly increasing global health expenditures and posing serious challenges to sustainable economic and social development. In China, these challenges are especially pronounced, placing new demands for adaptation and reform on the national health care system [4,5].

Globally, population ageing is accompanied by a high prevalence of chronic diseases and the widespread phenomenon of multimorbidity [6–8]. Multimorbidity refers to the coexistence of two or more chronic conditions, presenting specific combinatorial patterns where different chronic diseases exhibit interactive combinatorial effects – meaning the onset of one chronic condition may influence the risk of developing others [9–11]. Common chronic disease combinations include hypertension and diabetes, coronary heart disease and diabetes, osteoporosis and hypertension, among others [12]. Compared to patients with a single chronic condition, those with disease combinations often face higher risks of physical disability and more substantial economic and health care burdens, imposing significant pressure on population health and fiscal systems across nations and regions worldwide [13–15]. Consequently, delineating prevalent multimorbidity patterns and developing targeted interventions are essential steps toward mitigating risk, curbing costs and improving the efficiency of chronic-disease management [14].

The pathogenic mechanisms of multimorbidity often exhibit intertwined complex pathways, arising not only from interactions between chronic diseases but also from multilevel influences such as shared risk factors and social associated factors [6]. However, previous studies have predominantly focused on single or limited dimensions when exploring factors influencing multimorbidity in older adults, emphasising specific categories of influencing factors or individual chronic conditions. This approach lacks systematic investigation into the associated factors of multimorbidity across multiple dimensions [12,16–22]. Furthermore, existing evidence originates largely from high-income countries, with insufficient attention to developing regions facing heavier socioeconomic burdens [15]. China, as a representative developing country, faces a distinctive multimorbidity challenge marked by rapid population ageing, uneven health care resource distribution, and significant social transformation, all of which warrant in-depth investigation [23]. The health ecological model offers a multidimensional perspective with advantages for explaining diverse contributing factors [24]. Building on this framework, this study employs the health ecological model to investigate associated factors of multimorbidity in older adults across five dimensions, aiming to elucidate risk factors and inform targeted interventions.

Quality of Life (QoL) refers to individuals’ perceived health-related physical, psychological, and social functioning [25–28]. Multimorbidity not only severely compromises patients’ physical and mental health but also exerts profound negative impacts on their families and societal development [29–31]. Particularly for older adults, due to the complexity and refractory nature of multimorbidity, this population is more susceptible to the cumulative effects of disease resulting from multimorbidity, thereby adversely affecting their QoL [31,32]. Previous domestic and international research has explored the influence of individual characteristics, behaviours, and other factors on QoL through methods such as meta-analysis [4,25,28,33–35]. However, current studies on QoL in patients with multimorbidity still lack a systemic perspective. There has been insufficient comprehensive analysis of the interactions among multimorbidity, and a failure to deeply elucidate the associated factors of QoL across multiple levels [25,28]. Therefore, this paper categorises and analyses relevant variables according to macro-, meso-, and micro-level dimensions to systematically reveal the key influencing factors of QoL among older adults with multimorbidity.

When assessing the impact of chronic diseases on QoL among older adults, the EQ-5D-5L scale provides five response levels for each dimension, making the measurement more sensitive and enabling more detailed tracking of changes in individual health status [36,37]. Given China’s large population, regional and cultural diversity, and complex health needs, the EQ-5D-5L is a practical tool for assessing health status in such populations due to its concise structure and multidimensional design [38–40]. South China, especially Guangdong Province, serves as a microcosm of China’s rapid socioeconomic development alongside pronounced regional disparities. The characteristics of this region are also similar to those of numerous countries and regions globally facing comparable developmental challenges, thus lending representativeness to data derived from this area [41,42].

Based on the above background, this study conducted a survey on 2404 older adults aged 60 and above (including 1065 patients with multimorbidity) in Guangdong Province (Figure S1 in the **Online Supplementary Document**). It aimed to identify the influencing factors of multimorbidity based on the health ecological model, explore the combination patterns of chronic diseases and the association between the number of chronic diseases and QoL, and analyse the associated factors of QoL in patients with multimorbidity. Simultaneously, drawing on the WHO-ICOPE framework and adhering to a ‘people-centred’ concept, the study analyses the complex causes of multimorbidity among older adults from three dimensions – policy, community, and individual – proposes recommendations for constructing a comprehensive multimorbidity management system, and explores corresponding improvements and optimisations to care pathways. The findings aim to provide references for the precise prevention and intervention of chronic diseases and the improvement of overall QoL among older adults, thereby contributing to the construction of a Healthy China, while also offering a referenceable Chinese solution for older adults health governance in other developing countries and regions [43].

## METHODS

### Research design

From 1 September to 31 December 2023, a multi-stage random sample survey of Guangdong residents aged 60 years or older was carried out. One city was drawn from each of four provincial regions – the Pearl River Delta, eastern, western and northern Guangdong – according to economic level; within each city, a high-, middle- and low-income county or urban district was randomly selected, and two to three communities or villages were then chosen within each county. Of 3100 eligible residents (aged ≥60 years, local residency ≥6 months) invited, 2924 valid questionnaires were returned. After excluding 520 cases due to missing primary study variables, missing responses exceeding 20% in the questionnaire, severe data inconsistencies (*e.g*. age not matching birth year), or cognitive impairment identified by interviewers, 2404 participants remained, of whom 1065 were patients with multimorbidity, resulting in an effective response rate of 77.5%. Among them, if respondents self-reported having dementia-related diseases, or if field investigators assessed them as having cognitive impairment, they were defined as having cognitive impairment.

### Variable

#### Measurement of health status using the EQ-5D-5L scale

The Chinese EQ-5D-5L scale was used to measure health-related quality of life in this study [44]. It covers five dimensions – mobility, self-care, usual activities, pain/discomfort, and anxiety/depression – each rated on five levels: no, mild, moderate, severe, and extreme problems or inability. A utility index score was calculated using the China-specific value set (Table S1 in the **Online Supplementary Document**) [45], ranges from −0.391 (worst health) to 1.000 (best). Following earlier work [46], scores were classified into four categories: very poor, poor, good, and very good. The scale showed good internal consistency in this study (Cronbach’s α = 0.857).

#### Demographic and health-related variables

Demographic and health related data were collected through face-to-face interviews. Guided by the ecological model of health [47], variables were classified into five sequential layers: the policy environment (health insurance payment); living and working conditions (education level, per capita monthly household income, living area, region); the interpersonal network (living arrangement, marital status, caring for grandchildren, social participation); behavioural characteristics (smoking, drinking, fresh fruit intake, fresh vegetable intake, dietary balance, exercise, sleep quality); and individual characteristics (gender, age, BMI, number of chronic diseases) (Table S2 in the **Online Supplementary Document**).

Drawing on previous studies, the chronic-disease profile of Guangdong Province, and clinical expert advice [12,48–50], 24 chronic diseases diagnosed by doctors and self-reported by respondents were selected (Table S3 in the **Online Supplementary Document**), and each respondent’s total number of conditions was recorded.

### Data Analysis

#### Chronic disease combination patterns

The Apriori algorithm, a foundational method for association-rule mining introduced in 1994 by Agrawal and Srikant [51]. Association rules aim to uncover relationships among items in a data set, thereby revealing latent correlations. In this study, we used SPSS Modeler 18.0 (IBM Corp., Armonk, NY, USA) to analyse the data and applied the Apriori principle to explore combination patterns of chronic diseases, identifying combinations with strong inter-item associations. These patterns may inform the risk of additional chronic conditions based on existing disease profiles, offering valuable guidance for prevention and screening.

In association rule mining, support reflects the frequency of a rule, while confidence indicates its predictive strength. This study adopts a support threshold of 3% to ensure statistical stability while capturing common disease combinations. A confidence threshold of 50% was used to retain rules with meaningful predictive value. These parameters are consistent with those used in large-scale multimorbidity studies in China and abroad [52].

#### Factors influencing chronic diseases

Continuous variables were described using means (M) and standard deviations (SD), and group differences were tested using one-way analysis of variance (ANOVA). Qualitative data were expressed as frequencies (n) and percentages (%), and group differences were analysed using Pearson χ^2^ test. Variables included in the multivariate logistic regression met two criteria:

1) significant differences in ANOVA or χ^2^ tests

2) significant bivariate correlation with chronic disease count

Variables meeting these criteria were further included in the multivariate logistic regression model to explore their relationship with chronic diseases. The group without chronic diseases was set as the baseline, and the results were presented as odds ratios (OR) with 95% confidence intervals (CI). The significance level was set at *P* < 0.05. Data analyses were conducted using SPSS 27.0 software (IBM Corp., Armonk, NY, USA).

#### Association between chronic disease count and quality of life

Health utility values were used to reflect QoL, comparing the distribution of QoL data across different groups based on the number of chronic diseases. In the EQ-5D-5L, health utility values range from −0.391 to 1.000, with a truncation characteristic, which justifies the use of the Tobit regression model. Therefore, Tobit regression analysis was further performed using Stata 17.0 software (Stata Corp., College Station, TX, USA) to examine the relationship between the number of chronic diseases and QoL.

#### Factors influencing the quality of life in patients with multimorbidity

To further explore the factors influencing the QoL in patients with multimorbidity, this analysis focused on 1065 older adults with multimorbidity from the overall sample of 2404. Data analysis was performed using Stata 17.0 software. Since the EQ-5D-5L health utility values exhibited a skewed distribution, they were described using M(P25, P75), where M denotes the median, with P25 and P75 representing the 25th and 75th percentiles, respectively. The Wilcoxon rank-sum test was used for two-group comparisons, and the Kruskal-Wallis H test was used for multiple group comparisons. Therefore, statistically significant variables from the univariate analysis were considered as independent variables, with health utility values as the dependent variable, and were incorporated into a Tobit regression model to analyse the factors influencing the QoL in patients with multimorbidity. *P* < 0.05 was considered statistically significant.

## RESULTS

### Prevalence of chronic disease among older adults

Among the participants, 1065 individuals (44.3%) were affected by multimorbidity (two or more chronic diseases), 468 individuals (19.47%) had only one chronic disease, with hypertension being the most common (828 individuals, 34.4%), and malignant tumours being the least common (five individuals, 0.2%). For further details, refer to Table S3 in the **Online Supplementary Document**.

### Multimorbidity combination patterns based on association rules analysis

**Figure 1:**
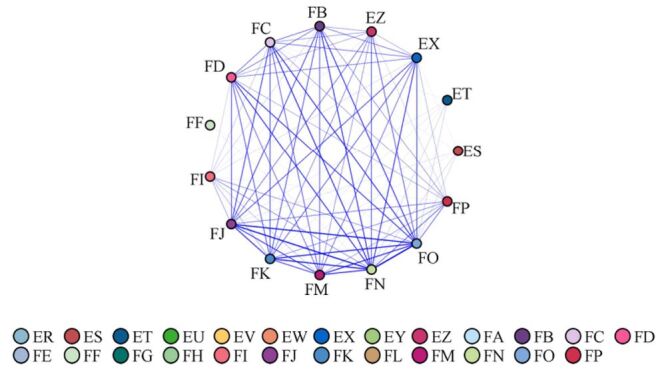
Network analysis of disease association rules for patients with multimorbidity. ER – coronary heart disease, ES – atherosis, ET – arrhythmia, EU – hypertension, EV – hyperlipidaemia, EW – chronic bronchi, EX – asthma, EY – cataract, EZ – glaucoma, FA – chronic gastritis, FB – chronic obstructive pulmonary disease, FC – chronic hepatitis, fatty liver, FD – chronic nephritis, FE – rheumatism or rheumatoid arthritis, FF – gout, FG – osteoporosis, FH – ischialgia, FI – stroke, FJ – Alzheimer disease, brain atrophy, Parkinson disease, FK – psycho, FL – diabetes, FM – hyperthyroidism, hypothyroidism, FN – cancer, FO – anaemia of chronic disease, iron deficiency anaemia, FP – others.

This study employed association rule analysis based on the Apriori algorithm to identify multimorbidity patterns using big data techniques. The minimum support value is set to 3.0%, the minimum confidence to 50%, and the maximum number of items to five, resulting in a network visualisation of effective association rules (**Figure 1**).

**Figure 2:**
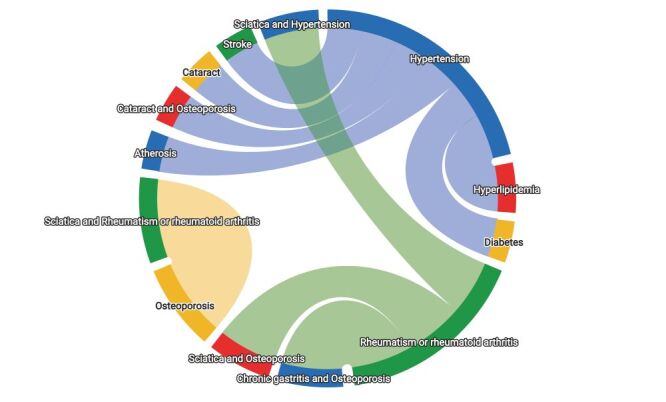
Top 10 disease-combination patterns in multimorbidity.

A total of 10 strong association rules were identified through analysis, six of which are related to hypertension, suggesting a higher likelihood of multimorbidity among individuals with hypertension. Additionally, there are five combinations of two chronic diseases, with the most common being ‘hypertension + hyperlipidemia’. For three chronic diseases, there are five combinations. The most frequent pattern was ‘rheumatic disease/rheumatoid arthritis + chronic gastritis + osteoporosis’ (**Figure 2**). Specific values can be found in Table S4 of the **Online Supplementary Document**.

### Factors influencing multimorbidity

#### Univariate analysis of factors associated with multimorbidity

Participants were categorised into three groups: no chronic disease, single chronic disease, and multimorbidity (**Table 1**). The results of the univariate analysis show that there is a statistically significant difference in the number of chronic diseases among older adults with different QoL (*P* < 0.05). Based on the health ecological model, stratification revealed significant differences in the number of chronic diseases among older adults concerning the following factors: gender, age, and BMI in the individual characteristics layer; dietary habits and sleep quality in the behavioural characteristics layer; living arrangement in the interpersonal network layer; education level, per capita monthly household income, living area and region in the living and working conditions layer; and health care payment methods in the macro policy layer, all of which show statistical significance (*P* < 0.05).

**Table 1:** Demographic characteristics and univariate analysis of factors influencing of multimorbidity among older adults (n = 2404)

Variable	Number of chronic diseases				*P*-value
	**0 (n = 871)**		**1 (n = 468)**	**≥2 (n = 1065)**	
**Quality of life, n (%)**					<0.001*
Very poor	49 (5.63)		25 (5.34)	130 (12.21)	
Poor	95 (10.91)		61 (13.03)	212 (19.91)	
Good	197 (22.62)		127 (27.14)	298 (27.98)	
Very good	530 (60.84)		255 (54.49)	425 (39.90)	
**Individual characteristics layer**					
**Gender, n (%)**					0.004*
Male	403 (46.27)		231 (49.36)	436 (40.94)	
Female	468 (53.73)		237 (50.64)	629 (59.06)	
**Age (years), mean (SD)**	69.56 (6.92)		70.59 (6.90)	71.46 (6.61)	<0.001*
**BMI (kg/m^2^), mean (SD)**	22.61 (3.05)		23.17 (3.17)	23.40 (3.50)	<0.001*
**Behavioural characteristics layer**					
**Smoke, n (%)**					0.017*
Yes	186 (21.35)		85 (18.16)	169 (15.87)	
Former	85 (9.76)		59 (12.61)	112 (10.52)	
Never	600 (68.89)		324 (69.23)	784 (73.61)	
**Drink, n (%)**					0.006*
Yes	192 (22.04)		94 (20.09)	179 (16.81)	
Former	81 (9.30)		41 (8.76)	135 (12.68)	
Never	598 (68.66)		333 (71.15)	751 (70.51)	
**Fresh fruit intake, n (%)**					<0.001*
Never	19 (2.18)		15 (3.21)	39 (3.66)	
Occasionally	393 (45.12)		199 (42.52)	435 (40.85)	
Often	212 (24.34)		136 (29.06)	352 (33.05)	
Always	247 (28.36)		118 (25.21)	239 (22.44)	
**Fresh vegetable intake, n (%)**					0.475
Never	6 (0.69)		1 (0.21)	6 (0.56)	
Occasionally	60 (6.89)		22 (4.70)	67 (6.29)	
Often	343 (39.38)		204 (43.59)	448 (42.07)	
Always	462 (53.04)		241 (51.50)	544 (51.08)	
**Dietary balance, n (%)**					<0.001*
Never	55 (6.31)		11 (2.35)	32 (3.00)	
Occasionally	202 (23.19)		92 (19.66)	199 (18.69)	
Often	356 (40.87)		223 (47.65)	491 (46.10)	
Always	258 (29.63)		142 (30.34)	343 (32.21)	
**Exercise (days), mean (SD)**	1.18(2.23)		1.45(2.51)	1.85(2.74)	<0.001*
**Sleep quality, n (%)**					<0.001*
Very good	285 (32.72)		102 (21.79)	149 (13.99)	
Good	438 (50.29)		239 (51.07)	577 (54.18)	
Poor	135 (15.50)		109 (23.29)	280 (26.29)	
Very poor	13 (1.49)		18 (3.85)	59 (5.54)	
**Interpersonal network layer**					
**Living arrangement, n (%)**					<0.001*
No children	29 (3.33)		5 (1.07)	7 (0.66)	
Absolutely empty nest	152 (17.45)		89 (19.02)	265 (24.88)	
Relatively empty nest	264 (30.31)		128 (27.35)	311 (29.20)	
Non-empty nest	426 (48.91)		246 (52.56)	482 (45.26)	
**Marital status, n (%)**					<0.001*
Married	681 (78.19)		362 (77.35)	772 (72.49)	
Single	21 (2.41)		8 (1.71)	5 (0.47)	
Divorced	10 (1.15)		4 (0.85)	13 (1.22)	
Widowed	159 (18.25)		94 (20.09)	275 (25.82)	
**Cares for grandchildren, n (%)**					0.037*
Always	89 (10.22)		43 (9.19)	109 (10.23)	
Often	86 (9.87)		65 (13.89)	108 (10.14)	
Occasionally	156 (17.91)		75 (16.02)	230 (21.60)	
Never	540 (62.00)		285 (60.90)	618 (58.03)	
**Social participation, mean (SD)**	1.76(1.42)		1.60(1.26)	1.57(1.23)	0.005*
**Living and working conditions layer**					
**Education level, n (%)**					<0.001*
No formal education	147 (16.88)		97 (20.73)	266 (24.98)	
Primary school	264 (30.31)		151 (32.26)	385 (36.15)	
Junior high school	209 (24.00)		120 (25.64)	222 (20.85)	
High school/Technical school	137 (15.73)		72 (15.38)	151 (14.18)	
Associate degree/Vocational college	46 (5.28)		15 (3.21)	26 (2.44)	
Bachelor’s degree or higher	68 (7.80)		13 (2.78)	15 (1.40)	
**Per capita monthly household income (CNY), n (%)**					0.006*
None	43 (4.94)		16 (3.42)	32 (3.00)	
<1000	115 (13.20)		71 (15.17)	177 (16.62)	
1000–3000	144 (16.53)		79 (16.88)	228 (21.41)	
3000–5000	281 (32.26)		144 (30.77)	329 (30.89)	
>5000	288 (33.07)		158 (33.76)	299 (28.08)	
**Region**					<0.001*
Pearl River Delta	408 (46.84)		107 (22.86)	248 (23.29)	
Western Guangdong	252 (28.93)		127 (27.14)	208 (19.53)	
Northern Guangdong	81 (9.30)		103 (22.01)	355 (33.33)	
Eastern Guangdong	130 (14.93)		131(27.99)	254(23.85)	
**Living area, n (%)**					<0.001*
City	465 (53.39)		235 (50.21)	439 (41.22)	
Rural	406 (46.61)		233 (49.79)	626 (58.78)	
**Policy environment layer**					
**Health insurance payment, n (%)**					0.002*
Self-pay	28 (3.21)		7 (1.50)	20 (1.88)	
Urban resident/employee insurance	816 (93.69)		457 (97.65)	1031 (96.81)	
Other	27 (3.10)		4 (0.85)	14 (1.31)	

BMI – body mass index, CNY – Chinese yuan, SD – standard deviation

*P-value is significant when ≤0.05

#### Multinomial logistics regression analysis of factors influencing multimorbidity

This study utilised a multinomial logistic regression model to investigate the factors influencing multimorbidity among older adults. The presence of diseases among older adults was set as the dependent variable (0 = no disease, 1 = single chronic disease, 2 = multimorbidity), with no disease as the reference group. Only variables with statistical significance in univariate analysis and bivariate correlation were included in the model. Quality of life, body mass index, sleep quality, living arrangement, region are the factors that influence older adults to suffer from a single chronic disease. Quality of life, age, body mass index, exercise, sleep quality, social participation, education level, per capita monthly household income, region are the influential factors for older adults to suffer from multimorbidity (**Table 2**).

**Table 2:** Results of multinomial logistic regression analysis of factors influencing of multimorbidity among older adults (n = 2404)

Variable	Simple chronic disease		Multimorbidity	
	**OR (95% CI)**	***P-*value**	**OR (95% CI)**	***P*-value**
Quality of life (reference: very good)				
*Very poor*	1.377 (0.787, 2.409)	0.262	4.683 (3.052, 7.187)	<0.001*
*Poor*	1.398 (0.943, 2.073)	0.096	2.750 (1.987, 3.805)	<0.001*
*Good*	1.444 (1.071, 1.945)	0.016*	1.957 (1.510, 2.537)	<0.001*
**Individual characteristics layer**				
Gender (reference: female)				
*Male*	1.235 (0.920, 1.657)	0.160	0.875 (0.677, 1.131)	0.308
Age (years)	1.020 (1.000, 1.040)	0.054	1.032 (1.015, 1.050)	<0.001*
BMI (kg/m^2^)	1.071 (1.030, 1.114)	<0.001*	1.117 (1.079, 1.155)	<0.001*
**Behavioral characteristics layer**				
Smoke (reference: never)				
*Yes*	0.757 (0.535, 1.070)	0.115	0.778 (0.574, 1.055)	0.106
*Former*	1.272 (0.843, 1.921)	0.252	1.102 (0.759, 1.600)	0.609
Exercise (days)	1.002 (0.951, 1.055)	0.950	1.056 (1.010, 1.104)	0.016*
Dietary balance (reference: always)				
*Never*	0.614 (0.298, 1.263)	0.185	0.577 (0.332,1.003)	0.051
*Occasionally*	1.086 (0.762, 1.548)	0.648	0.782 (0.577, 1.059)	0.112
*Often*	1.292 (0.975, 1.713)	0.075	1.059 (0.831, 1.351)	0.642
Sleep quality (reference: very poor)				
*Very good*	0.271 (0.122, 0.600)	0.001*	0.176 (0.087, 0.356)	<0.001*
*Good*	0.420 (0.195, 0.904)	0.027*	0.392 (0.200, 0.771)	0.007*
*Poor*	0.539 (0.246, 1.184)	0.124	0.465 (0.233, 0.929)	0.030*
**Interpersonal network layer**				
Living arrangement (reference: non-empty nest)				
*No children*	0.184 (0.038, 0.887)	0.035*	0.466 (0.136, 1.596)	0.224
*Absolutely*	0.764 (0.525, 1.113)	0.160	0.905 (0.657, 1.247)	0.542
*Relatively empty nest*	0.971 (0.729, 1.293)	0.841	1.272 (0.996, 1.625)	0.054
Marital status (reference: widowed)				
*Married*	1.102 (0.794, 1.528)	0.914	0.998 (0.758, 1.315)	0.990
*Single*	4.170 (0.968, 17.956)	0.057	0.678 (0.158, 2.910)	0.601
*Divorced*	1.279 (0.358, 4.565)	0.989	1.752 (0.649, 4.728)	0.268
Social participation	1.080 (0.971, 1.201)	0.158	1.099 (1.005, 1.202)	0.039*
**Living and working conditions layer**				
Education level (reference: Bachelor’s degree or higher)				
*No formal education*	1.592 (0.751, 3.376)	0.226	1.979 (0.975, 4.017)	0.059
*Primary school*	1.333 (0.657, 2.708)	0.426	2.115 (1.079, 4.147)	0.029*
*Junior high school*	1.581 (0.783, 3.194)	0.202	2.331 (1.189, 4.570)	0.014*
*High school/technical school*	1.562 (0.766, 3.185)	0.220	2.673 (1.357, 5.265)	0.004*
*Associate degree/vocational college*	1.078 (0.450, 2.579)	0.866	1.556 (0.694, 3.491)	0.284
Per capita monthly household income (CNY) (reference: >5000)				
*None*	0.959 (0.491, 1.874)	0.903	0.844 (0.476, 1.498)	0.563
*<1000*	1.057 (0.691, 1.616)	0.799	1.091 (0.755, 1.578)	0.642
*1000–3000*	1.138 (0.783, 1.655)	0.497	1.789 (1.299, 2.463)	<0.001*
*3000–5000*	1.071 (0.788, 1.457)	0.660	1.291 (0.985, 1.691)	0.064
Living area (reference: rural)				
*City*	1.235 (0.919, 1.658)	0.162	1.057 (0.821, 1.360)	0.669
Region (reference: Pearl River Delta)				
*Western Guangdong*	1.844 (1.272, 2.672)	0.001*	1.253 (0.914, 1.719)	0.161
*Northern Guangdong*	6.065 (3.665, 10.037)	<0.001*	8.390 (5.482, 12.841)	<0.001*
*Eastern Guangdong*	4.185 (2.805, 6.244)	<0.001*	4.310 (3.050, 6.090)	<0.001*

BMI – body mass index, CI – confidence interval, CNY – Chinese yuan, OR – odds ratio

*P-value is significant when ≤0.05.

### Relationship between the number of chronic diseases and quality of life

**Figure 3:**
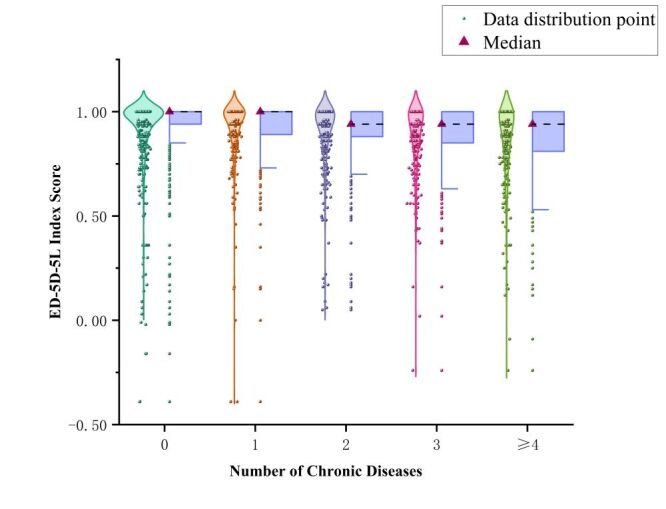
The comparison of EQ-5D-5L index score in different patients.

This study assessed the health utility values of older adults with different numbers of chronic diseases. The results showed that the median interquartile range (IQR) of health utility values for older adults without chronic diseases and those with a single chronic disease is 1. However, for those with two or more chronic diseases, the value dropped to 0.942 (**Figure 3**). This indicates that health utility values decline as the number of chronic diseases increases. Further analysis revealed that older adults with multimorbidity had a wider distribution of health utility values, indicating increased individual variation. Regression analysis using a Tobit model, with the number of chronic diseases as the independent variable and health utility value as the dependent variable, showed a negative correlation between the number of chronic diseases and health utility values (β = −0.014, *P* < 0.001). This indicates that as the number of chronic diseases increases, the QoL of older adults declines (Table S5 in the **Online Supplementary Document**).

### Factors associated with the quality of life of patients with multimorbidity

#### Quality of life and univariate analysis of patients with multimorbidity

Univariate analysis based on the health ecological model (Table S6 in the **Online Supplementary Document**) showed a median EQ-5D-5L utility of 0.942 (IQR 0.841–1.000) among participants with multimorbidity. Utility values differed significantly across individual, behavioural, and interpersonal domains. Higher scores were found in women; those who were overweight; respondents who smoked or drank alcohol; individuals who frequently consumed fresh vegetables and fruit, adhered to a balanced diet, exercised regularly, and reported good sleep; and those who were married, lived with family, engaged in social activities, held a college diploma or higher, often cared for grandchildren, resided in eastern Guangdong and urban areas. Utility declined with advancing age, a greater number of chronic conditions, and lower per capita monthly household income. Medical insurance type was not associated with health utility values.

#### Tobit regression analysis of factors influencing quality of life among patients with multimorbidity

Variables with statistical significance (*P* < 0.05) in the univariate analysis were selected as independent variables, with the health utility value of patients with multimorbidity as the dependent variable. A Tobit model was employed to explore factors associated with QoL in patients with multimorbidity. QoL among patients with multimorbidity was significantly associated with age, number of chronic diseases, drink, fresh fruit intake, dietary balance, exercise, sleep quality, social participation, education level, and region (**Table 3**).

**Table 3:** Tobit regression analysis of influencing factors of health utility value in patients with multimorbidity

Term	B	SE	*t*	*P*-value	95% CI
Constant (math.)	0.748	0.111	6.73	<0.001*	(0.530, 0.967)
**Age (years) (reference: 60–64)**					
65–69	−0.007	0.016	−0.42	0.671	(−0.039, 0.025)
70–74	−0.018	0.017	−1.04	0.300	(−0.052, 0.016)
75–79	−0.036	0.019	−1.87	0.062	(−0.073, 0.002)
≥80	−0.087	0.020	−4.34	<0.001*	(−0.126, -0.048)
**BMI kg/m^2^ (reference: <18.5)**					
18.5–23.9	0.007	0.019	0.35	0.723	(−0.030, 0.043)
24.0–27.9	0.023	0.020	1.16	0.248	(−0.016, 0.061)
≥28.0	0.003	0.024	0.12	0.905	(−0.044, 0.049)
**Number of chronic diseases (reference: 2)**					
3	−0.011	0.011	−1.01	0.313	(−0.033, 0.011)
>3	−0.029	0.011	−2.53	0.012*	(−0.051, −0.006)
**Smoke (reference: yes)**					
Former	−0.026	0.019	−1.41	0.158	(−0.063, 0.010)
Never	−0.002	0.013	−0.16	0.872	(−0.029, 0.024)
**Drink (reference: yes)**					
Former	−0.002	0.017	−0.14	0.887	(−0.037, 0.032)
Never	−0.029	0.014	−2.13	0.034*	(−0.055, −0.002)
**Fresh fruit intake (reference: never)**					
Occasionally	0.052	0.025	2.03	0.042*	(0.002, 0.102)
Often	0.064	0.026	2.41	0.016*	(0.012, 0.116)
Always	0.042	0.027	1.52	0.128	(−0.012, 0.095)
**Fresh vegetable intake (reference: never)**					
Occasionally	−0.045	0.064	−0.7	0.481	(−0.171, 0.080)
Often	−0.006	0.062	−0.1	0.922	(−0.127, 0.115)
Always	−0.011	0.062	−0.18	0.857	(−0.132, 0.110)
**Dietary balance (reference: never)**					
Occasionally	0.056	0.029	1.94	0.053	(−0.001, 0.112)
Often	0.044	0.028	1.56	0.118	(−0.011, 0.098)
Always	0.078	0.029	2.72	0.007*	(0.022, 0.135)
**Exercise (days) (reference: 0 d)**					
1–3 d	−0.039	0.016	−2.43	0.015*	(−0.070, −0.007)
>3 d	0.024	0.011	2.09	0.037*	(0.001, 0.046)
**Sleep quality (reference: very good)**					
Better	−0.034	0.014	−2.45	0.014*	(−0.062, −0.007)
Worse	−0.070	0.016	−4.51	<0.001*	(−0.101, −0.040)
Very bad	−0.161	0.024	−6.76	<0.001*	(−0.208, −0.114)
**Living arrangement (reference: no children)**					
Absolutely empty nest	0.020	0.077	0.26	0.798	(−0.131, 0.170)
Relatively empty nest	0.019	0.077	0.25	0.803	(−0.131, 0.169)
Non-empty nest	−0.017	0.076	−0.22	0.823	(−0.167, 0.133)
**Marital status (reference: married)**					
Single	−0.009	0.091	−0.10	0.923	(−0.187, 0.169)
Divorced	−0.011	0.042	−0.26	0.793	(−0.094, 0.072)
Widowed	0.008	0.011	0.67	0.503	(−0.015, 0.030)
**Cares for grandchildren (reference: always)**					
Often	−0.013	0.021	−0.65	0.513	(−0.054, 0.027)
Occasionally	−0.004	0.018	−0.22	0.830	(−0.039, 0.031)
Never	−0.024	0.016	−1.52	0.129	(−0.055, 0.007)
**Social participation (reference: no)**					
Yes	0.034	0.013	2.73	0.006*	(0.010, 0.059)
**Education level (reference: no formal education)**					
Primary school	0.028	0.013	2.20	0.028*	(0.003, 0.053)
Junior high school	0.017	0.015	1.10	0.271	(−0.013, 0.047)
High school/technical secondary school	−0.005	0.017	−0.28	0.781	(−0.038, 0.029)
College/higher vocational school	0.083	0.032	2.57	0.010*	(0.020, 0.146)
Bachelor’s degree or above	0.020	0.042	0.48	0.631	(−0.062, 0.102)
**Per capita monthly household income (CNY) (reference: none)**					
<1000	0.052	0.029	1.16	0.248	(−0.024, 0.091)
1000–3000	0.070	0.029	1.63	0.104	(−0.010, 0.104)
3000–5000	0.040	0.028	0.67	0.504	(−0.037, 0.075)
>5000	0.068	0.029	1.45	0.147	(−0.015, 0.099)
**Living area (reference: city)**					
Rural	0.002	0.012	0.17	0.866	(−0.022, 0.026)
**Region (reference: Pearl River Delta)**					
Western Guangdong	0.083	0.016	5.22	<0.001*	(0.052, 0.114)
Northern Guangdong	0.064	0.017	3.76	<0.001*	(0.031, 0.098)
Eastern Guangdong	0.132	0.017	7.89	<0.001*	(0.099, 0.165)

BMI – body mass index, CI – confidence interval, SE – standard error

*P-value is significant when ≤0.05.

## DISCUSSION

As population ageing intensifies, the prevalence of chronic diseases among middle-aged and older adults continues to rise. This study covers more than 24 chronic diseases and, guided by the health ecological model, systematically investigated the prevalence of multimorbidity, risk factors, multimorbidity patterns, and factors affecting patients’ QoL.

### Prevalence and cross-country comparison of multimorbidity among older adults

This study found that the prevalence of multimorbidity among Chinese older adults aged 60 and above is 44.3%, more than twice that of those with a single chronic condition (19.5%). Another nationally representative study reports that the morbidity rate for multiple diseases among Chinese middle-aged and older adults aged 45 and above reaches 54.3%. Compared to the research by Ma et al., this study provides data specifically focused on the population aged 60 and above [53,54]. In addition, although there are certain differences between the results of this study and those of Bangladesh (56.4%), which is also a developing country, reflecting shared public health challenges related to chronic disease management in developing settings [55]. Compared to developed nations, Canada’s disease prevalence rate is significantly higher at 80.84%, which may be attributed to differences in geriatric age stratification and the more comprehensive disease diagnosis and reporting systems in high-income countries [56]. Notably, the prevalence rate observed in this study substantially exceeds that of Nepal, the least developed country, at 13.96% [57]. This disparity may reflect China’s comparatively advanced health infrastructure and diagnostic capabilities.

Among at least 24 chronic diseases, hypertension has the highest prevalence rate, a conclusion consistent with those drawn in other Asian countries [58], the United Kingdom, and other Western countries [59–61]. High salt intake is one of the main risk factors leading to hypertension [62]. The traditional Chinese diet has a high salt intake. Although sodium intake has decreased over the past 30 years, it still exceeds the recommended level [63]. Unhealthy dietary patterns among older adults contribute to the persistent high prevalence of hypertension [64]. The WHO recommends this intervention as one of the most cost-effective strategies to mitigate the burden of non-communicable diseases worldwide [65]. Therefore, it is essential to enhance hypertension prevention and management through dietary modifications and improved nutritional strategies.

### Multimorbidity patterns and targeted prevention strategies

This study also utilises association rule mining to analyse big data, uncovering multimorbidity disease cluster patterns. The findings reveal several key commonalities in these multimorbidity patterns. First, hypertension frequently co-occurs within multimorbidity patterns, particularly in conjunction with hyperlipidaemia and diabetes, indicating a high coexistence of cardiovascular and metabolic risk factors among patients with multimorbidity. This finding supports the widely recognised hypothesis that cardiovascular and metabolic diseases tend to cluster [60,66,67]. In response, several authoritative Chinese health care institutions have jointly developed and issued the “Chinese Guidelines for the Prevention of Cardiovascular and Metabolic Diseases”, providing targeted lifestyle intervention recommendations for patients. Second, rheumatoid arthritis commonly coexists with chronic gastritis and osteoporosis, highlighting the systemic impact of chronic inflammation. Arthritis, hearing impairment, and osteoporosis are regarded as classic degenerative conditions that significantly impair the daily functional capacity among older adults [68]. Degenerative conditions arising from ageing and impaired tissue repair merit particular attention. The high prevalence and diverse combinations of multimorbidity underscore the necessity for enhanced prevention and management strategies, emphasising holistic and integrated chronic disease management approaches. Special attention should be given to developing specific prevention and treatment protocols for highly comorbid disease clusters. Furthermore, after diagnosing cardiovascular-metabolic or degenerative single chronic diseases, older patients should undergo regular health screenings, with a focus on preventing and controlling other related chronic conditions to reduce the overall incidence of multimorbidity.

### Factors associated with multimorbidity and practical implications

At the individual characteristic level, age and body mass index (BMI) are significant factors influencing the prevalence of multimoribidity, with advanced age and overweight status serving as key risk factors [69]. Regarding behavioural characteristic, this study indicates that older adults with better sleep quality exhibit a lower risk of multimoribidity compared to those with poorer sleep quality. Similar findings from other regional studies also demonstrate an association between poor sleep quality and increased risks of dyslipidaemia and hyperglycaemia [70]. Additionally, the World Health Organization reports that adequate sleep not only plays a crucial role in maintaining healthy body weight, such as optimal BMI, but also indirectly reduces the risk of multimoribidity through various mechanisms [71]. Unexpectedly, frequent physical activity was positively associated with multimorbidity. This may reflect reverse causality, where individuals with existing conditions increase physical activity based on medical advice rather than physical activity being a causal factor.

The prevalence of multimorbidity is closely associated with interpersonal network status. Empty nest status was not significantly associated with multimorbidity but was related to the prevalence of single chronic conditions. Specifically, childless older adults exhibit lower risk of single chronic disease compared to those cohabiting with children. This may be due to intergenerational conflicts arising from differing perspectives, which can adversely affect mental and physical health, thereby increasing disease risk. Alternatively, it might reflect higher frailty levels among parous women vs nulliparous women [72,73]. Nonetheless, based on existing research, it is advisable to pay attention to the potential negative impacts of empty nest status, such as providing targeted home-based assistance and emergency support [74]. Additionally, findings indicate that higher levels of social engagement are associated with an increased risk of multimorbidity, possibly because patients diagnosed with their first chronic disease tend to participate more actively in community activities as part of health education efforts. Future studies should employ longitudinal designs to better establish the causal sequence between social participation and the onset of chronic diseases.

Furthermore, at the level of living and working conditions, older adults with higher socioeconomic status (education, income) were less likely to experience multimorbidity [75]. Variations in socioeconomic status (income, education) influence health outcomes through multiple pathways, primarily affecting individuals’ access to health care resources and the development of protective factors such as healthy lifestyle behaviours [76]. Higher socioeconomic groups tend to have greater access to medical services and health literacy, whereas lower socioeconomic groups may face limited health care accessibility, leading to the adoption of unhealthy lifestyle practices [77]. In light of these findings, policymakers should implement targeted interventions and allocate resources to ensure that patients with multimorbidity, particularly those in socioeconomically disadvantaged populations, can access appropriate health care services and health education. Enhancing health awareness and behavioural practices among older adults is essential for effective prevention and management of multimorbidity [78].

Older adults in northern and eastern Guangdong face a higher risk of multimorbidity, likely due to regional socioeconomic disparities and widespread labour migration. Compared with the more developed Pearl River Delta, these areas see many working-age adults migrate for jobs, weakening local health support for older adults. Evidence shows that such migration increases health risks among low-income and older rural populations [79], raising the likelihood of multimorbidity. To address this issue, policy efforts should focus on strengthening county medical consortiums, reallocating health care resources to primary care institutions, and expanding chronic disease screening. In addition, smart health monitoring devices connected to township health centres can help identify high-risk individuals and support timely home visits and interventions.

### Interplay between quality of life and multimorbidity

In addition to incorporating factors across the hierarchical levels of the health ecological model, the study findings indicate that QoL is influencing multimorbidity. Conversely, multimorbidity significantly impacts QoL by affecting individuals’ psychological, physiological, and social mechanisms [7,80]. Health utility values were used to assess QoL. Participants with no or one chronic disease showed utility scores near 1.000, while those with two or more conditions had significantly lower scores, averaging 0.942. Furthermore, Tobit regression analysis confirmed a significant negative correlation between the number of chronic diseases and health utility values, indicating a continuous decline in QoL with increasing multimorbidity [81]. The detrimental impact of multimorbidity on QoL often surpasses that of other factors [82]. As the number of chronic conditions increases, cumulative health risks and mortality rise sharply. Early intervention and effective management are therefore critical to preserving QoL.

### Optimising quality of life in patients with multimorbidity through associated factors and strategic interventions

Focusing on patients with multimorbidity, this study investigates the key associated factors of health-related QoL across macro, meso, and micro levels to identify comprehensive intervention strategies.

At the macro-level, no statistically significant differences were observed between health insurance reimbursement models and patient QoL, warranting further exploration of underlying factors [83–85]. Multimorbidity, often co-occurring with other diseases, significantly exacerbates the economic burden on patients, posing substantial challenges [86]. Although some provinces and municipalities in China have implemented policies addressing the needs of multimorbid patients, there remains room for policy enhancement [87]. Given the reliance of multimorbid patients on continuous outpatient care, future research should consider designing targeted health insurance policies. Additionally, findings indicate that patients in the economically developed Pearl River Delta region experience markedly lower QoL compared to those in western, northern, and eastern Guangdong, suggesting potential side effects of urbanisation, such as environmental pollution, higher living costs, and elevated health expectations, on patient well-being [88,89].

Transitioning to the meso-level, social networks play a pivotal role. The findings indicate that older adults with higher levels of social engagement generally exhibit elevated health utility values. Active social participation is consistently associated with improved psychological well-being and contributes to enhancing the overall resilience of patients’ physical and mental health [90,91].

Focusing on the micro-level, individual patient characteristics and behavioral patterns are associated with QoL. The health utility values of patients with multimorbidity decline with age, with advanced age serving as a decisive factor [92]. There is a negative correlation between health utility and the number of chronic diseases an individual has: an increased burden of chronic illness significantly diminishes QoL. Additionally, certain individual behaviours are found to be associated with better QoL, including frequent consumption of fresh fruits, a balanced diet, adequate physical activity, high-quality sleep, and extensive social participation. The study also reveals that QoL improves with higher socioeconomic status, consistent with previous research [93]. For instance, older adults with higher educational attainment demonstrate better disease knowledge and self-management capabilities, whereas those with lower education levels face more barriers. Notably, the findings indicate a positive effect of alcohol consumption on patients’ QoL, which may be attributed to regional cultural characteristics. In the Pearl River Delta and eastern and western Guangdong regions, alcohol is often integrated into family, clan, and business activities to enhance social emotional support and subjective well-being [94–96]. However, it is important to recognise that multiple studies have demonstrated health risks associated with excessive alcohol intake; therefore, moderate consumption is still recommended [97,98].

In addressing the QoL challenges faced by older adults with multimorbidity, this proposal draws upon the WHO-ICOPE framework. Emphasising a people-centred approach, it advocates for a multidimensional strategy encompassing policy, community, and individual levels to establish an integrated and precise model of health management services for multimorbidity. First, it recommends enhancing the level of medical insurance coverage for multimorbidity in China. Governments at all levels should enhance policy support by establishing specialised health care coverage for multimorbidity patients, particularly through flexible reimbursement stacking mechanisms. Priority should be given to rural and low-income populations, with adjusted stacking rules and reimbursement ratios based on disease severity to alleviate financial burdens. Second, guided by the ICOPE service guidelines, community organisations and members should be encouraged to participate in delivering health and personalised social care services, ensuring continuity and integration across screening, assessment, intervention, management, and treatment of various chronic conditions among older adults. Finally, individuals, under the guidance of national policies and community health education initiatives, should enhance their health literacy and leverage digital intelligence technologies to strengthen self-management. These measures are expected not only to improve overall health outcomes but also to significantly enhance QoL, thereby effectively addressing the challenges posed by multimorbidity.

### Strengths and limitations

This study concentrates on the issue of multimorbidity, utilising data from on-site team surveys to conduct a series of in-depth analyses that ensure research coherence. Initially, it delineates the current landscape of multimorbidity; subsequently, it explores the patterns of disease combinations to reveal the complexity of the prevailing multimorbidity framework; then, it examines the influencing factors of multimorbidity to provide a scientific basis for preventive strategies. The study further investigates the relationship between the number of chronic conditions and QoL, followed by a detailed analysis of the associated factors affecting QoL among patients with multimorbidity, offering valuable insights for enhancing their overall well-being.

Although the research findings offer significant insights, several limitations must be acknowledged. First, this study employs a cross-sectional design, which only allows for the identification of associations between multimorbidity, QoL, and related factors, without establishing causality. Future longitudinal cohort studies are recommended to elucidate the causal relationships among multimorbidity, QoL, and influencing determinants. Second, the data primarily rely on self-reported information and oral interviews, which are susceptible to recall bias and subjective distortions, potentially leading to reporting bias. It is advisable for future research to incorporate clinical examination data as a reference standard to enhance the accuracy and validity of the conclusions.

## CONCLUSIONS

Multimorbidity has now surpassed single chronic diseases as the most common health issue among older adults in southern China, with cardiovascular-metabolic diseases such as hypertension and hyperlipidaemia being the most prevalent. Multimorbidity is closely associated with lower quality of life and is related to factors such as age, body mass index, physical activity, sleep, social participation, education level, income, and region. Regression analysis confirmed that drink, fruit intake, and dietary balance significantly predict quality of life. To alleviate this burden, efforts should be made at the macro level to improve medical insurance reimbursement and public health policies, at the meso level to expand community-based comprehensive care, and at the micro level to promote healthy lifestyles and active social participation, providing evidence-based pathways to improve the quality of life for China’s ageing population.

## Additional material


Online Supplementary Document
