# Supplementary material for: Prevalence, combination patterns, and quality of life factors of multimorbidity among older adults in southern China based on the health ecological model
Source: J Glob Health. 2025 Jul 25;15:04215. doi: 10.7189/jogh.15.04215 (PMC12290435; doi:10.7189/jogh.15.04215)
Supplement: Online Supplementary Document [file jogh-15-04215-s001.pdf]

Supplement to: Long C, Huang J, Liu D, Liu C, Wu M, Wu H, Deng J, Zhang Y, Shi L, Cui Y. Prevalence, combination patterns, and quality of life factors of multimorbidity among older adults in southern China based on the health ecological model. J Glob Health. 2025;15:04215.

Contents

Figure S1 in the Online Supplementary Document. **Graphical abstract** 1

Table S1 in the Online Supplementary Document. **The health utility values corresponding to the dimension scores of the Chinese version of the ED-5D-5L scale** 1

Table S2 in the Online Supplementary Document. **Definition of variable assignments in health ecology** 1

Table S3 in the Online Supplementary Document. **Prevalence of chronic diseases among older adults** 3

Table S4 in the Online Supplementary Document. **Patterns of disease combinations for patients with multimorbidity** 4

Table S5 in the Online Supplementary Document. **Tobit regression analysis of number of chronic diseases and health utility value** 6

Table S6 in the Online Supplementary Document. **Comparison of health utility values in patients with multimorbidity** 6

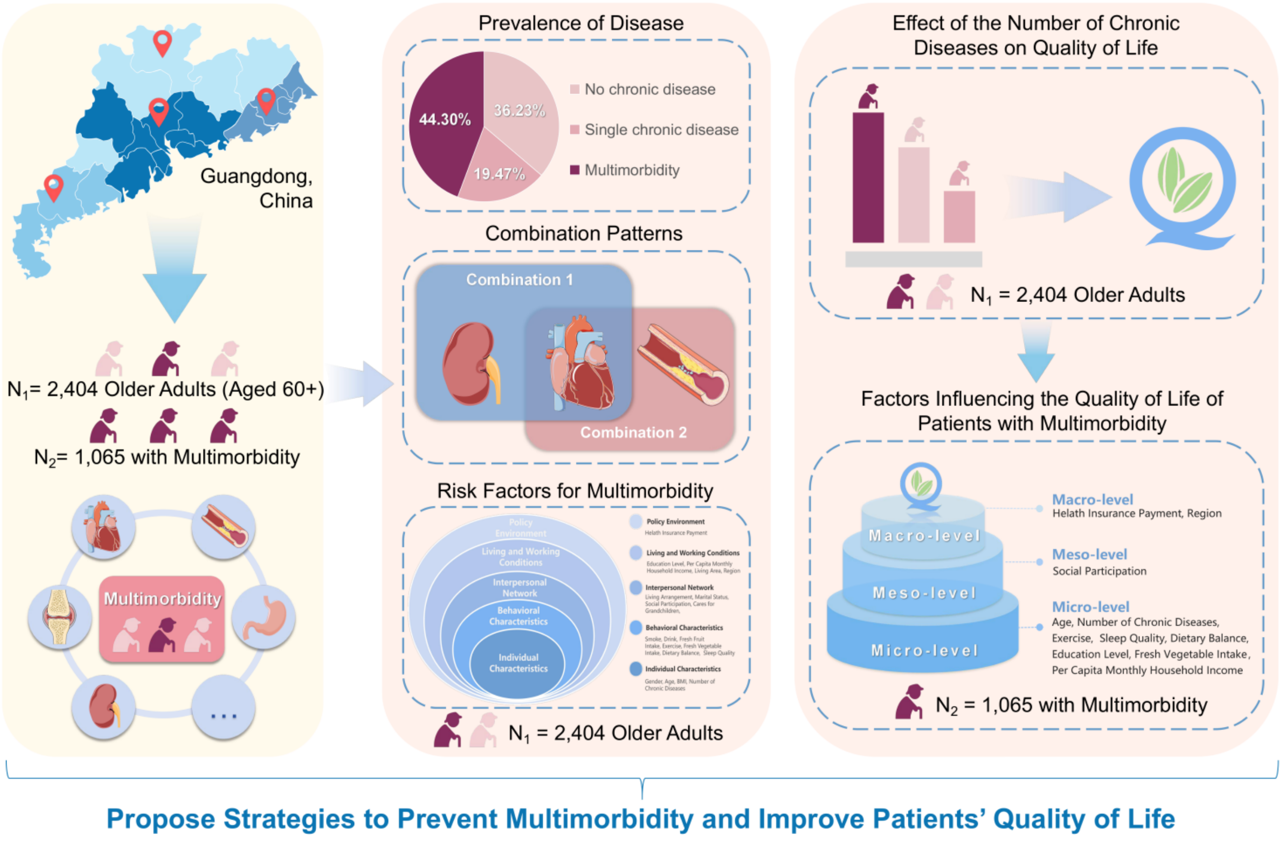

Figure S1 in the **Online Supplementary Document. Graphical abstract**

Table S1 in the **Online Supplementary Document. The health utility values corresponding to the dimension scores of the Chinese version of the ED-5D-5L scale**

Level	MO	SC	UA	PD	AD
1	0.000	0.000	0.000	0.000	0.000
2	0.066	0.048	0.045	0.058	0.049
3	0.158	0.116	0.107	0.138	0.118
4	0.287	0.210	0.194	0.252	0.215
5	0.345	0.253	0.233	0.302	0.258

The scoring formula is as follows: U = 1 - (MOn + SCn + UAn + PDn + ADn). Here, n = 1, 2, 3, 4, 5, represents the severity level for each dimension.

Table S2 in the **Online Supplementary Document. Definition of variable assignments in health ecology**

Variable settings	Variables	Value of variables
Quality of Life variable	Quality of Life	Very poor =1, Poor = 2, Good = 3, Very good = 4
Policy Environment Layer	Insurance	Self-Pay = 1, Urban Resident/Employee Insurance = 2, Other = 3
Living and Working Conditions Layer	Education Level	No formal education = 1, Primary school = 2, Junior high school = 3, High school/Technical school = 4, Associate degree/Vocational college = 5, Bachelor's degree or higher = 6
	Per Capita Monthly Household Income	None = 1, <1000 = 2, 1000-3000 = 3, 3000-5000 = 4, >5000 = 5
	Living Area	City = 1, Rural = 2
	Region	Pearl River Delta = 1, Western Guangdong = 2, Northern Guangdong = 3, Eastern Guangdong = 4
Interpersonal Network	Living Arrangement	No children = 1, Absolutely empty nest = 2, Relatively empty nest = 3, Non-empty nest = 4
	Marital Status	Married = 1, Single = 2, Divorced = 3, Widowed = 4
	Cares for Grandchildren	Always = 1, Often = 2, Occasionally = 3, Never = 4
	Social Participation	Number of social activities in the past month, Continuous variable
Individual Characteristics Layer	Gender	Male = 1, Female = 2
	Age	Years, Continuous variable
	BMI	Kg/m^2, Continuous variable
Behavioral Characteristics Layer	Smoke	Yes = 1, Former = 2, Never = 3
	Drink	Yes = 1, Former = 2, Never = 3
	Fresh Fruit Intake	Never = 1, Occasionally = 2, Often = 3, Always = 4
	Fresh Vegetable	Never = 1, Occasionally = 2, Often = 3, Always = 4

	Intake	
	Dietary Balance	Never = 1, Occasionally = 2, Often = 3, Always = 4
	Exercise	Number of days of moderate exercise per week, Continuous variable
	Sleep Quality	Very good = 1, Good= 2, Poor = 3, Very poor = 4

Table S3 in the **Online Supplementary Document. Prevalence of chronic diseases among older adults**

**Chronic Disease**	**Number of Case**	**Types of Chronic Diseases[N(%)]**				**Multimorbidity**
	**N(%)**	**1**	**2**	**3**	**⩾4**	**N(%)**
All participants	2404(100.00)	468(19.47)	421(17.51)	323(13.44)	321(13.35)	1065(44.30)
Hypertension	828(34.40)	186(22.46)	215(25.97)	198(23.91)	229(27.66)	642(77.54)
Rheumatism	522(21.70)	63(12.07)	129(24.71)	127(24.33)	203(38.89)	459(87.93)
Osteoporosis	397(16.50)	28(7.05)	72(18.14)	121(30.48)	176(44.33)	369(92.95)
Diabetes	238(9.90)	35(14.71)	62(26.05)	52(21.85)	89(37.39)	203(85.29)
Chronic Gastritis	251(10.40)	33(13.15)	50(19.92)	65(25.90)	103(41.04)	218(86.85)
Cataract	231(9.60)	12(5.19)	35(15.15)	69(29.87)	115(49.78)	219(94.81)
Coronary Heart Disease	222(9.20)	19(8.56)	47(21.17)	48(21.62)	108(48.65)	203(91.44)
Ischialgia	188(7.80)	12(6.38)	39(20.74)	52(27.66)	85(45.21)	176(93.62)
Chronic Bronchitis	151(6.20)	13(8.61)	38(25.17)	31(20.53)	69(45.70)	138(91.39)
Hyperlipidemia	149(6.20)	7(4.70)	25(16.78)	45(30.20)	72(48.32)	142(95.30)
Gouty Arthritis	132(5.50)	7(5.30)	20(15.15)	29(21.97)	76(57.58)	125(94.70)
Atherosis	112(4.70)	6(5.36)	18(16.07)	0(0.00)	88(78.57)	106(94.64)
Arrhythmia	111(4.60)	5(4.50)	8(7.21)	17(15.32)	81(72.97)	106(95.50)

Stroke	79(3.30)	7(8.86)	19(24.05)	17(21.52)	36(45.57)	72(91.14)
Glaucoma	59(2.50)	2(3.39)	8(13.56)	13(22.03)	36(61.02)	57(96.61)
Asthma	43(1.80)	2(4.65)	12(27.91)	5(11.63)	24(55.81)	41(95.35)
Chronic Obstructive Pulmonary Disease	40(1.70)	5(12.50)	6(15.00)	5(12.50)	24(60.00)	35(87.50)
Chronic Nephritis	39(1.60)	4(10.26)	7(17.95)	6(15.38)	22(56.41)	35(89.74)
Chronic Hepatitis	36(1.50)	3(8.33)	2(5.56)	7(19.44)	24(66.67)	33(91.67)
Hyperthyroidism / Hypothyroidism	32(1.30)	2(6.25)	11(34.38)	9(28.13)	10(31.25)	30(93.75)
Emotional or Mental Problems	24(1.00)	1(4.17)	3(12.50)	5(20.83)	15(62.50)	23(95.83)
Senile Dementia	17(0.70)	2(11.76)	1(5.88)	4(23.53)	10(58.82)	15(88.24)
Anemia of Chronic Disease	7(0.30)	0(0.00)	0(0.00)	1(14.29)	6(85.71)	7(100.00)
Malignant Tumor	5(0.20)	2(40.00)	3(60.00)	0(0.00)	0(0.00)	3(60.00)
Other	113(4.70)	12(10.62)	15(13.27)	15(13.27)	71(62.83)	101(89.38)

Table S4 in the **Online Supplementary Document. Patterns of disease combinations for patients with multimorbidity**

Consequent	Antecedent	Support (%)	Confidence (%)
Hypertension	Hyperlipidemia	6.198	75.839
Hypertension	Diabetes	9.900	65.126
Rheumatism/rheumatoid arthritis	Chronic gastritis and Osteoporosis	3.120	64.000
Rheumatism/rheumatoid arthritis	Ischialgia and Osteoporosis	4.201	62.376
Osteoporosis	Ischialgia and Rheumatism or	4.201	62.376

	rheumatoid arthritis		
Hypertension	Atherosis	4.659	59.821
Hypertension	Cataract and Osteoporosis	3.369	59.259
Hypertension	Cataract	9.609	58.442
Hypertension	Stroke	3.286	58.228
Rheumatism/rheumatoid arthritis	Ischialgia and Hypertension	3.037	57.534
Hypertension	Chronic gastritis and Osteoporosis	3.120	57.333
Hypertension	Cataract and Rheumatism or rheumatoid arthritis	3.286	56.962
Hypertension	Arrhythmia	4.617	56.757
Hypertension	Coronary heart disease	9.235	55.856
Hypertension	Coronary heart disease and Rheumatism or rheumatoid arthritis	3.161	55.263
Hypertension	Gout	5.491	54.545
Osteoporosis	Ischialgia	7.820	53.723
Rheumatism or rheumatoid arthritis	Ischialgia	7.820	53.723
Osteoporosis	Ischialgia and Hypertension	3.037	53.425
Rheumatism or rheumatoid arthritis	Chronic gastritis and Hypertension	4.409	50.943
Osteoporosis	Cataract and Rheumatism or rheumatoid arthritis	3.286	50.633
Rheumatism or rheumatoid arthritis	Gout	5.491	50.000

Hypertension	Chronic gastritis and Rheumatism or rheumatoid arthritis	4.493	50.000

Table S5 in the **Online Supplementary Document.** Tobit regression analysis of number of chronic diseases and health utility value

**Term**	***B***	***SE***	***t***	***P***	**95% CI**
Number of chronic diseases	-0.014	0.002	-8.03	<0.001	(-0.017, -0.011)

SE – standard error, CI – confidence interval

**P*-value is significant when ≤0.05.

Table S6 in the **Online Supplementary Document. Comparison of health utility values in patients with multimorbidity**

**Variable**	**Population** [n(%)]	**ED-5D-5L Index Score** [M(P_{25}, P_{75})]	***Z/H***	***P***
**Total**	1065(100.0)	0.942(0.841, 1.000)		
**Gender**				
Male	436(40.9)	0.942 (0.862, 1.000)	-0.603	0.547
Female	629(59.1)	0.942 (0.840, 1.000)		
**Age (Years)**				
60-64	114(10.7)	1.000 (0.893, 1.000)	54.228	＜0.001
65-69	366(34.2)	0.942 (0.889,1.000)		
70-74	268(25.1)	0.942(0.862,1.000)		
75-79	173(16.2)	0.897 (0.813,1.000)		
≥80	144(13.5)	0.876 (0.717,1.000)		
**BMI（Kg / m^2）**				
＜18.5	74(6.9)	0.942 (0.822, 1.000)	26.333	<0.001

18.5-23.9	561(52.7)	0.942 (0.815, 1.000)		
24.0-27.9	340(31.9)	0.942 (0.893, 1.000)		
⩾28.0	90(8.5)	0.942 (0.844, 1.000)		
**Number of Chronic Diseases**				
2	421(39.5)	0.942 (0.876, 1.000)	12.201	0.002
3	323(30.3)	0.942 (0.848, 1.000)		
＞3	321(30.1)	0.942 (0.813, 1.000)		
**Smoke**				
Yes	169(15.9)	0.942 (0.862, 1.000)	6.727	0.035
Former	112(10.5)	0.916 (0.780, 1.000)		
Never	784(73.6)	0.942 (0.862, 1.000)		
**Drink**				
Yes	179(16.8)	0.942 (0.876, 1.000)	6.754	0.034
Former	135(12.7)	0.934 (0.824, 0.942)		
Never	751(70.5)	0.942 (0.840, 1.000)		
**Fresh Fruit Intake**				
Never	39(3.7)	0.907 (0.744, 0.942)	73.748	<0.001
Occasionally	435(40.8)	0.893 (0.813, 1.000)		
Often	352(33.1)	0.942 (0.893, 1.000)		
Always	239(22.4)	0.942 (0.893, 1.000)		
**Fresh Vegetable Intake**				
Never	6(0.6)	0.893 (0.729, 1.000)	15.801	0.001
Occasionally	67(6.3)	0.897 (0.734, 0.955)		
Often	448(42.1)	1.000 (0.862, 1.000)		
Always	544(51.1)	0.942 (0.842, 1.000)		
**Dietary Balance**				
Never	32(3.0)	0.862 (0.734, 0.986)	38.194	<0.001
Occasionally	199(18.7)	0.893 (0.813, 1.000)		

Often	491(46.1)	0.942 (0.840, 1.000)		
Always	343(32.2)	0.942 (0.889, 1.000)		
**Exercise (Days)**				
0 Day	682(64.0)	0.942 (0.824, 1.000)	15.437	<0.001
1-3 Days	107(10.0)	0.942 (0.824, 1.000)		
>3 Days	276(25.9)	0.942 (0.889, 1.000)		
**Sleep Quality**				
Very good	149(14.0)	1.000 (0.942, 1.000)	90.996	<0.001
Better	577(54.2)	0.942 (0.862, 1.000)		
Worse	280(26.3)	0.897 (0.813, 1.000)		
Very bad	59(5.5)	0.824 (0.666, 0.942)		
**Living Arrangement**				
No children	7(0.7)	0.942 (0.813, 0.942)	12.193	0.007
Absolutely empty nest	265(24.9)	0.934 (0.840, 1.000)		
Relatively empty nest	311(29.2)	0.942 (0.831, 1.000)		
Non-empty nest	482(45.3)	0.942 (0.862, 1.000)		
**Marital Status**				
Married	772(72.5)	1.000 (0.862, 1.000)	23.440	<0.001
Single	5(0.5)	0.942 (0.827, 0.942)		
Divorced	13(1.2)	1.000 (0.654, 1.000)		
Widowed	275(25.8)	1.000 (0.813, 1.000)		
**Cares for Grandchildren**				
Always	109(10.2)	0.942 (0.862, 1.000)	38.504	<0.001
Often	108(10.1)	0.978 (0.894, 1.000)		
Occasionally	230(21.6)	1.000 (0.876, 1.000)		
Never	618(58.0)	0.942 (0.824, 1.000)		
**Social Participation**				
No	191(17.9)	0.894 (0.748, 0.955)	-5.105	<0.001

Yes	874(82.1)	0.942 (0.862, 1.000)		
**Education Level**				
No formal education	266(25.0)	0.893 (0.768, 0.942)	69.350	<0.001
Primary school	385(36.2)	0.942 (0.862, 1.000)		
Junior high school	222(20.8)	1.000 (0.892, 1.000)		
High school / technical secondary school	151(14.2)	1.000 (0.894, 1.000)		
College / higher vocational school	26(2.4)	0.947 (0.896, 1.000)		
Bachelor degree or above	15(1.4)	0.942 (0.731, 1.000)		
**Per Capita Monthly Household Income (CNY)**				
None	32(3.0)	0.893 (0.786, 1.000)	32.513	＜0.001
<1000	177(16.6)	0.893 (0.813, 1.000)		
1000-3000	228(21.4)	0.942 (0.862, 1.000)		
3000-5000	329(30.9)	0.942 (0.844, 1.000)		
>5000	299(28.1)	0.951 (0.876, 1.000)		
**Living area**				
City	439(41.2)	0.952 (0.882, 1.000)	-5.619	<0.001
Rural	626(58.8)	0.942 (0.822, 1.000)		
**Region**				
Pearl river delta	248 (23.29)	0.820 (0.768, 1.000)	182.534	<0.001
Western Guangdong	208 (19.53)	0.876 (0.824, 1.000)		
Northern Guangdong	355 (33.33)	0.876 (0.827, 1.000)		
Eastern Guangdong	254 (23.85)	0.972 (0.906, 1.000)		
**Health Insurance Payment**				
Medical insurance for Urban Workers	216(20.3)	0.942 (0.862, 1.000)	3.061	0.548

Medical insurance for urban and rural residents	798(74.9)	0.942 (0.840, 1.000)		
Commercial Health Insurance	19(1.8)	0.942 (0.715, 0.951)		
Other means (free medical care, social assistance)	12(1.1)	0.947 (0.854, 1.000)		
All at your own expense	20(1.9)	0.942 (0.834, 1.000)		

M - Mean

**P*-value is significant when ≤0.05.
